# Sensory descriptors, hedonic perception and consumer’s attitudes to Sangiovese red wine deriving from organically and conventionally grown grapes

**DOI:** 10.3389/fpsyg.2013.00896

**Published:** 2013-11-29

**Authors:** Ella Pagliarini, Monica Laureati, Davide Gaeta

**Affiliations:** ^1^Department of Food, Environmental and Nutritional Sciences (DeFENS), Università degli Studi di MilanoMilano, Italy; ^2^Dipartimento di Economia Aziendale, Università degli Studi di VeronaVerona, Italy

**Keywords:** odor, taste, organic wine, consumer expectation, sensory, willingness to pay

## Abstract

In recent years, produce obtained from organic farming methods (i.e., a system that minimizes pollution and avoids the use of synthetic fertilizers and pesticides) has rapidly increased in developed countries. This may be explained by the fact that organic food meets the standard requirements for quality and healthiness. Among organic products, wine has greatly attracted the interest of the consumers. In the present study, trained assessors and regular wine consumers were respectively required to identify the sensory properties (e.g., odor, taste, flavor, and mouthfeel sensations) and to evaluate the hedonic dimension of red wines deriving from organically and conventionally grown grapes. Results showed differences related mainly to taste (sour and bitter) and mouthfeel (astringent) sensations, with odor and flavor playing a minor role. However, these differences did not influence liking, as organic and conventional wines were hedonically comparable. Interestingly, 61% of respondents would be willing to pay more for organically produced wines, which suggests that environmentally sustainable practices related to wine quality have good market prospects.

## INTRODUCTION

The sensory analysis of wine has always given rise to interest both in the scientific community and among consumers. Wine is tightly tied to psychological aspects besides being purely sensory. There have been many studies carried out on different aspects connected with wine tasting such as the cognitive and perceptual processes that characterize wine expertise. Wine-tasting expertise involves advanced discriminative and descriptive abilities with respect to wine. While the basis of wine expertise remains unknown, differences in performance between experts and novices are relatively clear (Lawless, 1984; Noble et al., 1987; Solomon, 1990; Hughson and Boakes, 2002; Zucco et al., 2011). Wine-tasting experts such as sommeliers have obviously a greater sensory ability than inexperienced novices, but their knowledge of wine may sometimes lead them to misperception of the product (Pangborn et al., 1963; Morrot et al., 2001). Pangborn et al. (1963) and Morrot et al. (2001) carried out experiments in which white wines were colored to obtain rosé and red wines, respectively. Pangborn et al. (1963) found that such a modification led wine experts but not novices to judge the product as sweeter than colorless controls. Similarly, Morrot et al. (2001) showed that wine experts described the white wine with the characteristics of a red wine.

While there are several studies on wine perception, little is known about sensory characteristics of wines deriving from organically and conventionally grown grapes. Organic agriculture is a production management system that promotes and enhances biodiversity, biological cycles, and soil biological activity. The primary goal of organic agriculture is to minimize all forms of pollution and to avoid the use of synthetic fertilizers and pesticides, thus optimizing the health and productivity of soil, plants, animals, and humans.

In recent years, consumers have become increasingly concerned by the effects of conventional agricultural production practices on both human and environmental health. As a consequence, production obtained from organic farming methods has been rapidly growing in developed countries. This may be explained as organic food adequately meets all requirements for quality, genuineness, and healthiness (Forbes et al., 2009). Recent evidence has also shown an increase of the related literature, even though studies are still few in number. The studies comparing foods derived from organic and conventional growing systems focused mainly on three topics: nutritional value, sensory quality, and food safety (Bourn and Prescott, 2002).

Relative to the nutritional value of wine, its antioxidant activity and benefit on health were addressed (Renaud and De Lorgeril, 1992), showing that phenolic compounds are natural anti-inflammatory and efficient scavengers of free radicals (Akçay et al., 2004).

As to the sensory quality of food products, reports indicate that organic and conventional fruits and vegetables may differ on a variety of sensory aspects; however, findings are inconsistent (Bourn and Prescott, 2002). Therefore, the assumption of organic food having a better taste may be explained by the consumer’s expectation of a healthier and safer product evoked by the label “organic food” (Deliza and MacFie, 1996). Indeed, expectations greatly influence subject responses (see e.g., Dalton et al., 1997).

Few studies compared sensory properties of wines derived from organically and conventionally grown grapes. Moyano et al. (2009) for instance, examined the aroma profile of sherry wines that had been cultivated conventionally and organically and found that organic wines had a sensory profile similar to that of the conventional ones, but lower odor intensity. The same findings were reported by Dupin et al. (2000), who examined German wines and found that organic products tended to be less aromatic than conventional ones.

“Sangiovese” (*Vitis vinifera* L.) is the most widely consumed Italian wine. It is used to produce prestigious Tuscan wines such as Chianti and Brunello di Montalcino. To our knowledge no studies are available on Sangiovese red wine sensory quality. Thus, the main aim of this work is to identify and describe the sensory properties, such as odor, taste, flavor, and mouthfeel sensations, that characterize organically and traditionally grown Romagna Sangiovese red wines. Also, as sensory properties greatly influence food preference, the hedonic dimension of organic and conventional wines was investigated.

## MATERIALS AND METHODS

### WINES

The red wines evaluated in the present study were produced from ripe grapes from *Vitis Vinifera* Sangiovese harvested in September 2007 and 2008 in the region of Faenza (Italy). The grapes were derived from two different farms located in adjacent areas and subjected to similar environmental conditions. For both vintages, one farm produced grapes according to organic techniques whereas the other adopted conventional agricultural techniques. At variance from conventionally cultivated grapes neither insecticides nor synthetic fertilizers were used in organic agriculture during the growth.

All wines were produced following the same process according to PDO (Protected Designation of Origin) specifications. Wines were analyzed 6 months after they were bottled. Three bottles from the organic and three from the traditional production of vintage 2007 were randomly selected to be used for sensory analysis and the same procedure was used for vintage 2008.

## SENSORY ANALYSIS

### PARTICIPANTS

Descriptive analysis of wines: 12 assessors (seven women and five men) aged on average 27.0 ± (SD) 3.5 years (range 23–35 years) were selected. They were trained to evaluate organic and conventional wines from vintages 2007 and 2008.

Hedonic test of wines: a second group of 100 (50 women and 50 men) regular red wine consumers (inexpert individuals with no formal wine training) aged on average 32.1 ± (SD) 9.6 years (range, 20–60 years) participated.

The participants were students and employees of the University of Milan, who reported liking red wine and consuming it more than twice a month. None of the participants had previous or present taste or smell disorders. The study was in accordance with the Declaration of Helsinki. The protocol was approved by the Institutional Ethics Committee at the study site. Informed consent was obtained from all subjects.

#### Descriptive analysis

Descriptive analysis (Lawless and Heymann, 1998; ISO International Organization for Standardization, 2003) was used to identify and quantify the sensory properties of organic and conventional wines from two successive vintages.

Training phase: subjects were trained over a period of 2 months. During the first part of the training, assessors tasted Romagna Sangiovese wines and set up a list of descriptors that characterized the wines. To do so, assessors wrote down as many terms as they could to describe the sensory characteristics fully. Assessors agreed through panel discussion on what terms were relevant, and arrived at definitions for each term. At this stage, a reference product was provided in order to help the assessors to understand each term.

Evaluation phase: after training was completed, the panel evaluated the two wines (organic vs. conventional) in triplicate. Judges were instructed to drink and swallow each sample and rate the intensity of each attribute using a nine-point scale (1 = absence of the sensation and 9 = maximum intensity). The sessions were performed on the same day (with a minimum 2-h break between the sessions) at the sensory laboratory of the Department of Food, Environmental and Nutritional Sciences (DeFENS, Università degli Studi di Milano) designed in accordance with ISO guidelines (ISO International Organization for Standardization, 2007). Data acquisition was done using Fizz v2.31 software (Biosystèmes, Couternon, France). Assessors were asked not to smoke, eat or drink anything, except water, at least 1 h before the tasting sessions. For each sample, judges received a 30 ml sample served in glasses coded with a three-digit number and covered with a Petri dish to avoid the escape of volatile components. Participants were provided with mineral water and unsalted crackers to clean their mouth between tastings. Wines were served at 18 ± 1°C. Presentation orders were systematically varied over assessors and replicates in order to balance the effects of serving order and carryover (MacFie et al., 1989).

#### Consumer’s preference and attitude toward wine consumption

Since the sensory properties of a food are among the primary determinants of food preference and choice, we also investigated the hedonic qualities of organic and conventional Romagna Sangiovese wines. For this purpose, the two wines under study, organic and conventional from vintage 2008, were evaluated along with four other Romagna Sangiovese wines from the same vintage produced according to conventional agriculture techniques, which were purchased in local wineries and were comparable for price category to those under study. Due to practical constraints (i.e., no availability of wine), the wines from vintage 2007 were not included in the hedonic evaluation.

Consumers were invited to take part in a hedonic test carried out at the DeFENS sensory laboratory. Each participant received a series of six wines (20 ml for each product) served in glasses coded with three-digit numbers and covered with Petri dishes. For each sample, participants were instructed to drink and swallow the wine and rate the degree of liking using a seven-point hedonic scale (with 1 = extremely disliked and 7 = extremely liked; Lawless and Heymann, 1998). Consumers were asked to drink mineral water and to eat a piece of unsalted cracker to clean their mouth between tastings. Also, they were asked not to smoke, eat or drink anything, except water, 1 h before the tasting session. Data were collected using Fizz v2.31g software program (Biosystemes, Couternon, France). Wines were evaluated under standard light conditions at a temperature of 18 ± 1°C. In order to balance the effects of serving order and carryover, the presentation order of the wines was randomized. After the liking test, the subjects were asked a few questions about their wine consumption habit and organic wine purchase likelihood.

## RESULTS

### DESCRIPTIVE ANALYSIS

The panel generated a total of 12 descriptors that characterize the sensory profile of the wines: four odor descriptors (fruity, spicy, woody, and vanilla), two taste descriptors (sour and bitter), three flavor descriptors (fruity, spicy, and woody) and three mouthfeel sensations (astringent, alcohol, and body). Complete definitions and standard products for all descriptors are listed in **Table 1**.

**Table 1:** List of the 12 sensory descriptors of Romagna Sangiovese PDO wines with their relevant definitions and reference standards.

Descriptor	Definition	Reference standard
**Odor**
Fruity	Characteristic odor of a combination of blueberry, raspberry, and blackberry perceived by means of the sense of smell (orthonasal perception)	Infusion (24 h, 4°C) of 12 blueberries, two raspberries, and one blackberry in 0.5 l of red table wine
Spicy	Characteristic odor of a combination of spices (cinnamon and clove) perceived by means of the sense of smell (orthonasal perception)	Infusion (24 h, 4°C) of 16 cloves and one cinnamon stick in 0.5 l of red table wine
Vanilla	Characteristic odor of vanilla perceived by means of the sense of smell (orthonasal perception)	Commercial liquid vanilla odorant (2 ml) dissolved in 0.5 l of red table wine
Woody	Characteristic odor of toasted wood perceived by means of the sense of smell (orthonasal perception)	Guaiacol in red table wine (2 ppb)
**Taste**
Sour	One of the basic tastes, caused by solution of acidic compounds perceived in the oral cavity	Anhydrous citric acid (2 g) in 0.7 l of red table wine
Bitter	One of the basic tastes, caused by solution of bitter compounds perceived in the oral cavity	Caffeine (0.8 g) in 0.5 l of red table wine
**Flavor**
Fruity	Characteristic odor of a combination of blueberry, raspberry, and blackberry perceived by means of the sense of smell during swallowing (retronasal perception)	Infusion (24 h, 4°C) of 12 blueberries, two raspberries, and one blackberry in 0.5 l of red table wine
Spicy	Characteristic odor of a combination of spices (cinnamon and clove) perceived by means of the sense of smell during swallowing (retronasal perception)	Infusion (24 h, 4°C) of 16 cloves and one cinnamon stick in 0.5 l of red table wine
Woody	Characteristic odor of toasted wood perceived by means of the sense of smell during swallowing (retronasal perception)	Guaiacol in red table wine (2 ppb)
**Mouthfeel**
Astringent	Mouth dryness caused by tannins and perceived in the oral cavity	Dissolve 1.5 g of tannin in 750 ml of red table wine
Alcohol	Characteristic heat/burning sensation perceived in the oral cavity	Mix 40 ml of 95% ethyl alcohol with 500 ml of red table wine
Body	Characteristic perceived in the oral cavity, due to the friction among the molecules in a liquid, that gives to it a limited fluidity and mobility	Mix 6 ml of glycerol with 1 l of red table wine

**FIGURE 1:**
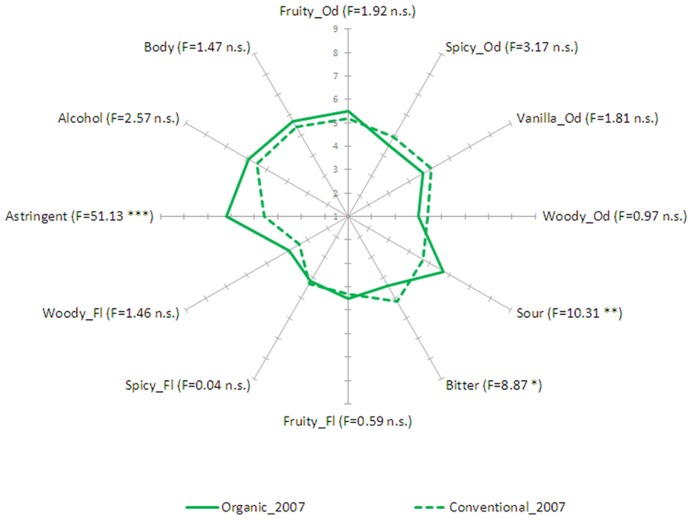
**Descriptive analysis results: mean values for each sensory descriptor by method of production (organic vs. conventional) for vintage 2007.** For each descriptor the relevant significance is reported (***p < 0.001, **p < 0.01, *p < 0.05).

**FIGURE 2:**
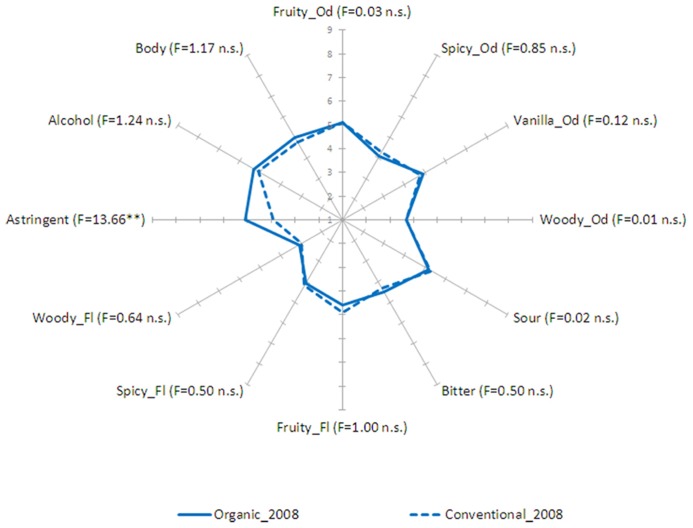
**Descriptive analysis results: mean values for each sensory descriptor by method of production (organic vs. conventional) for vintage 2008.** For each descriptor the relevant significance is reported (**p < 0.01).

Mean intensity ratings of organic and conventional wines are reported in **Figures 1** and **2**. Intensity data for each sensory descriptor from the two vintages were analyzed separately through ANOVA with *Wines* (organic vs. conventional), *Judges*, *Replicates *(rep 1 vs. rep 2 vs. rep3) as factors. Relative to vintage 2007, *Wines* were significantly different for sour taste (*F* = 10.31, *p* < 0.01), bitter taste (*F* = 8.87, *p* < 0.05) and astringency (*F* = 51.13, *p* < 0.001). *Post-hoc* comparison using the Bonferroni test (*p* < 0.05) showed that organic wine was perceived as having a higher intensity of sour taste, and astringent sensation but lower bitter taste. Differences between the two wines from vintage 2008 concerned only astringency (*F* = 13.66, *p* < 0.01), with organic wine having a higher intensity. The effect of *Judges* was significant (*p* < 0.05), which is expected because individuals can of course have different sensitivities to the different descriptors. This effect can seldom be changed by training (Lea et al., 1997). Also, data analysis showed that *F* values for *Replicates *and interactions between *Wines and Judges*, *Judges and Replicates* and *Wines and Replicates* were not significant (*p* < 0.05) for nearly all the attributes. These results indicated that the mean scores for each wine given by the assessors for each attribute could be assumed to be satisfactory estimates of the sensory profile of the samples (i.e., good panel reliability).

### STUDY OF CONSUMER PREFERENCE AND ATTITUDE TOWARD WINE CONSUMPTION

Mean hedonic ratings and standard errors for organic and conventional Romagna Sangiovese wines are reported in **Table 2**. Data analysis by means of one-way ANOVA showed significant differences (*F* = 2.42, *p* < 0.05) between wines for liking ratings. *Post-hoc* comparison using the Bonferroni test (*p* < 0.05) showed that organic and conventional wines from vintage 2008 were not significantly different and showed liking ratings comparable to other commercial wines (Sangiovese A, B, and C).

**Table 2:** Mean hedonic ratings (±STDERR) for organic and conventional Romagna Sangiovese wines from vintage 2008 and other four commercial Romagna Sangiovese wines from conventional agricultural techniques (Sangiove A–D).

Wines (*F* = 2.42; *p* < 0.05)	Hedonic rating
Sangiovese A	4.2^a^ ± 0.3
Conventional 2008	4.4^a^ ± 0.3
Organic 2008	4.5^a^ ± 0.3
Sangiovese B	4.8^ab^ ± 0.3
Sangiovese C	4.9^ab^ ± 0.3
Sangiovese D	5.3^b^ ± 0.3

*Mean hedonic ratings with different superscripts are significantly different according to Bonferroni test (*p* < 0.05).*

The same subjects involved in the hedonic study were also asked to answer a few questions about their attitude toward wine consumption (see, **Table 3**). About 59% of the subjects were habitual red wine consumers. The largest part (85%) of the wine used was mostly for home consumption. Wine is purchased at retail shops (59%) and most of the consumers are used to spending no more than 7 euros for a bottle of wine. Finally, it is interesting to note that when asked about the purchase of organically produced wine, 61% of them declared they would be willing to pay more for such product.

**Table 3:** Results from the questionnaire related to wine consumption habit and organic wine purchase intention.

Question	Answer (%)	Items
How would you define yourself?	59	Habitual wine consumer (2 or more times a month)
	41	Occasional wine consumer (less than twice a week)

Wine purchase is mainly destined to…	85	Home consumption
	15	Restaurant consumption

Where do you usually buy wine?	12	Wine shops
	59	Retail shops
	29	Wineries

How much do you usually pay for a bottle of wine?	3	Less than 3 euros
	19	Between 3 and 5 euros
	49	Between 5 and 7 euros
	28	Between 7 and 10 euros
	2	More than 10 euros

Would you be willing to pay an extra charge for an organically produced wine?	23	Yes, less than 10%
	34	Yes, between 10 and 20%
	4	Yes, between 20 and 30%
	0	Yes, more than 30%
	39	No

## DISCUSSION

The present study investigated the sensory and hedonic qualities of red wines derived from organically and conventionally grown grapes. The examined wines were Romagna Sangiovese red wines. The descriptive analysis identified specific olfactory properties that characterize these wines, namely fruity, spicy, vanilla, and woody odors and flavors. Odor is a relevant sensory attribute of food, as well as of wines, which lead consumer’s preference and choice. Also, the quality and specificity of each wine are associated in most cases with a specific odorant.

This study has shown that the organic and conventional wines differed marginally in the intensity of sensory descriptors. Only the properties of taste and mouthfeel sensations distinguished the two types of wine, whereas odor and flavor seemed to play a minor role. Organic wine from vintage 2007 was perceived as more sour and astringent but less bitter than its conventional counterpart, whereas differences between wines from vintage 2008 concerned only astringency.

In addition, the differences between wines did not influence liking, as organic and conventional wines were hedonically comparable. This means that consumers are not able to discriminate among organic and conventional wines from a hedonic point of view. One reason relates to their lack of formal training in sensory evaluation, which leads them only to detect major differences among products with less sensitivity to more subtle differences. It may be assumed that differences in liking could have been perceived between organic and conventional wines from vintage 2007, which showed larger differences in the intensity of some sensory qualities (i.e., bitter taste, sour taste and astringency) than wines from vintage 2008. Unfortunately, this hypothesis could not be verified, as wines from vintage 2007 were not included in the hedonic comparison. Nevertheless, self-reported comments by the participants suggest that even though the organic wine from vintage 2007 showed a high intensity of sourness and astringency, it was judged equally liked as its conventional counterpart.

The issue of comparing the hedonic qualities of organically and conventionally produced food has been tackled by various authors with respect to different food products, e.g., yogurt (Laureati et al., 2013), cheese (Napolitano et al., 2010a), meat (Napolitano et al., 2010b), and beer (Caporale and Monteleone, 2004). Interestingly, in these studies the liking of organic and conventional products has been evaluated under different information conditions: the blind condition (i.e., consumers taste and judge the product without any kind of information); the expected condition (i.e., consumers do not taste the product and judge it only on the basis of written or visual information); and the informed condition (i.e., consumers taste and judge the product after having read written information and/or seen an image). The main outcome of these studies is that organic products are liked more than their conventional counterparts but only in informed conditions, namely when consumers knew that they were to taste an organic food. Thus, it would seem that organic products are liked more because of the “healthier” connotation they have in the consumer’s mind rather than for an actual preference based on perceptual attributes. Also, the influence of information about organic production on consumers’ food preferences and expectations is especially evident in the case of consumers who are more interested in and proactive for “sustainable” products (Laureati et al., 2013). This suggests that expectation plays an important role for food consumption, since it may improve or degrade the perception of a product, even before it is tasted (Deliza and MacFie, 1996; Dalton et al., 1997). In this respect, it should be pointed out that the Sangiovese wines used in the present study were evaluated under blind conditions, without any information concerning production method. Thus, consumers’ liking derives mainly from the mere sensory perception of the wines without any pre-conceived ideas due to their knowledge about the product.

Finally, an interesting result is that most of the consumers declared themselves willing to pay more for organically produced wines. This result is in line with the finding of a recent study by Lockshin and Corsi (2012) who reported that consumers in European countries as well as in the United States, New Zealand and Australia are willing to pay more for organic wines mainly for health and environmental reasons but also because consumers are interested in helping producers who adopt these innovations. Of course cognitive factors as personal expectancies – addressed above – have room. Therefore, a greater predisposition to pay an additional charge for organic wine may be due to specific consumer’s attitude and involvement in sustainability issues.

In conclusion, the present study evidenced the sensory properties that characterize red wines from organically and conventionally grown grapes. The differences detected from a quantitative point of view are only marginal, and do not seem to have an impact on consumer’s hedonic perception. A limitation of this study may be that only two vintages of one grape variety of organic and conventional wines were considered. Further research is needed to clarify this aspect. In this context, future perspectives of study should deal with the study of sensory and hedonic qualities of wine, which are undoubtedly the strongest determinants of consumer’s expectations and play a key role in consumer’s purchase attitude. This aspect seems to be particularly relevant for wines deriving from organically and conventionally grapes since environmentally sustainable practices related to wine quality seem to have good market prospects.

## Conflict of Interest Statement

The authors declare that the research was conducted in the absence of any commercial or financial relationships that could be construed as a potential conflict of interest.

## References

[B1] Akçay Y. D. Yıldırım H. K. Güvenç U. Sözmen E. Y. (2004). The effects of consumption of organic and nonorganic red wine on low-density lipoprotein oxidation and antioxidant capacity in humans. *Nutr. Res.* 24 541–554 10.1016/j.nutres.2004.04.004

[B2] Bourn D. Prescott J. (2002). A comparison of the nutritional value, sensory qualities, and food safety of organically and conventionally produced foods. *Crit. Rev. Food Sci.* 42 1–34 10.1080/10408690290825439 11833635

[B3] Caporale G. Monteleone E. (2004). Influence of information about manufacturing process on beer acceptability. *Food Qual. Pref.* 15 271–278 10.1016/S0950-3293(03)00067-3

[B4] Dalton P. Wysocki C. J. Brody M. J. Lawley H. J. (1997). The influence of cognitive bias on the perceived odor, irritation and health symptoms from chemical exposure. *Int. Arch. Occup. Environ. Health* 69 407–417 10.1007/s004200050168 9215927

[B5] Deliza R MacFie H. J. H. (1996). The generation of sensory expectation by external cues and its effect on sensory perception and hedonic ratings: a review. *J. Sens. Stud.* 7 253–277 10.1111/j.1745-459X.1996.tb00036.x

[B6] Dupin I. Schlich P. Fischer U. (2000). “Differentiation of wines produced by organic or conventional viticulture according to their sensory profiles and aroma composition,” in *Proceedings of Proceedings 6th International Congress on Organic Viticulture,* eds H. Willer and U. Meier (Bad Dürkheim, D: Print-Online) 245–251

[B7] Forbes S. L. Cohen D. A. Cullen R. Wratten S. D. Fountain J. (2009). Consumer attitudes regarding environmentally sustainable wine: an exploratory study of the New Zealand marketplace. *J. Clean. Prod.* 17 1195–1199 10.1016/j.jclepro.2009.04.008

[B8] Hughson L. Boakes R. (2002). The knowing nose: the role of knowledge in wine expertise. *Food Qual. Pref.* 13 463–472 10.1016/S0950-3293(02)00051-4

[B9] ISO International Organization for Standardization. (2003). *Sensory Analysis – Methodology – General Guidance for Establishing a Sensory Profile* (ISO 13299:2003). Geneva, Switzerland

[B10] ISO International Organization for Standardization. (2007). *Sensory Analysis – General Guidance for the Design of Test Rooms* (ISO 8589:2007). Geneva, Switzerland

[B11] Laureati M. Jabes D. Russo V. Pagliarini E. (2013). Sustainability and organic production: how information influences consumer’s expectation and preference for yogurt. *Food Qual. Pref.* 30 1–8 10.1016/j.foodqual.2013.04.002

[B12] Lawless H. T. (1984). Flavor description of white wine by expert and non-expert wine consumers. *J. Food Sci.* 49 120–123 10.1111/j.1365-2621.1984.tb13686.x

[B13] Lawless H. T. Heymann H. (1998). “Descriptive analysis,” in *Sensory Evaluation of Food: Principles and Practices* eds Lawless H. T. Heymann H. (New York: Chapman & Hall) 341–372

[B14] Lea P. Næs T. Rødbotten M. (1997). “Further aspects of design and modeling,” in *Analysis of Variance for Sensory Data* (Chichester: John Wiley & Sons Ltd) 20–21

[B15] Lockshin L. Corsi A. M. (2012). Consumer behavior for wine 2.0: a review since 2003 and future directions. *Wine Econ. Policy* 1 2–23 10.1016/j.wep.2012.11.003

[B16] MacFie H. J. H. Bratchell N. Greenhoff K. Vallis L. V. (1989). Designs to balance the effect of order of presentation and first order carry-over effects in hall tests. *J. Sens. Stud.* 4 129–148 10.1111/j.1745-459X.1989.tb00463.x

[B17] Morrot G. Brochet F. Dubourdieu D. (2001). The color of odors. *Brain Lang.* 79 309–320 10.1006/brln.2001.2493 11712849

[B18] Moyano L. Zea L. Villafuerte L. Medina M. (2009). Comparison of odor-active compounds in sherry wines processed from ecologically and conventionally grown pedroximenez grapes. *J. Agric. Food Chem.* 57 968–973 10.1021/jf802252u 19146368

[B19] Napolitano F. Braghieri A. Piasentier E. Favotto S. Naspetti S. Zanoli R. (2010a). Cheese liking and consumer willingness to pay as affected by information about organic production. *Food Qual. Pref.* 18 280–286 10.1017/S0022029910000130 Epub 2010 Mar 3 20196900

[B20] Napolitano F. Braghieri A. Piasentier E. Favotto S. Naspetti S. Zanoli R. (2010b). Effect of information about organic production on beef liking and consumer willingness to pay. *Food Qual. Pref.* 21 207–212 10.1016/j.foodqual.2009.08.007 20196900

[B21] Noble A. Arnold R. A. Buechsenstein J. Leach E. J. Schmidt J. O. Stern P. M. (1987). Modification of a standardized system of wine aroma terminology. *Am. J. Enol. Vitic.* 38 143–146

[B22] Pangborn R. Berg H. Hansen B. (1963). The influence of color on discrimination of sweetness in dry table-wine. *Am. J. Psychol.* 76 492–495 10.2307/1419795

[B23] Renaud S De Lorgeril M. (1992). Wine, alcohol, platelets, and the French paradox for coronary heart disease. *Lancet* 339 1523–1526 10.1016/0140-6736(92)91277-F 1351198

[B24] Solomon G. (1990). Psychology of novice and expert wine talk. *Am. J. Psychol.* 105 495–517 10.2307/1423321

[B25] Zucco G. Carassai A. Baroni M. R. Stevenson R. J. (2011). Labeling, identification, and recognition of wine-relevant odorants in expert sommeliers, intermediates, and untrained wine drinkers. *Perception* 40 598–607 10.1068/p6972 21882722

